# C-reactive protein independently predicts survival in pancreatic neuroendocrine neoplasms

**DOI:** 10.1038/s41598-021-03187-x

**Published:** 2021-12-09

**Authors:** Anna Nießen, Simon Schimmack, Marta Sandini, Dominik Fliegner, Ulf Hinz, Magdalena Lewosinska, Thilo Hackert, Markus W. Büchler, Oliver Strobel

**Affiliations:** 1 Department of General, Visceral and Transplantation Surgery, Heidelberg University Hospital, Im Neuenheimer Feld 420, 69120 Heidelberg, Germany; 2 Division of Visceral Surgery, Department of General Surgery, Medical University of Vienna, Vienna, Austria

**Keywords:** Cancer, Medical research, Oncology, Risk factors

## Abstract

Pancreatic neuroendocrine neoplasms (pNEN) are highly variable in their postresection survival. Determination of preoperative risk factors is essential for treatment strategies. C-reactive protein (CRP) has been implicated in the pathogenesis of pNEN and shown to be associated with survival in different tumour entities. Patients undergoing surgery for pNEN were retrospectively analysed. Patients were divided into three subgroups according to preoperative CRP serum levels. Clinicopathological features, overall and disease-free survival were assessed. Uni- and multivariable survival analyses were performed. 517 surgically resected pNEN patients were analysed. CRP levels were significantly associated with relevant clinicopathological parameters and prognosis and were able to stratify subgroups with significant and clinically relevant differences in overall and disease-free survival. In univariable sensitivity analyses CRP was confirmed as a prognostic factor for overall survival in subgroups with G2 differentiation, T1/T2 and T3/T4 tumour stages, patients with node positive disease and with and without distant metastases. By multivariable analysis, preoperative CRP was confirmed as an independent predictor of postresection survival together with patient age and the established postoperative pathological predictors grading, T-stage and metastases. Preoperative serum CRP is a strong predictive biomarker for both overall and disease free survival of surgically resected pNEN. CRP is associated with prognosis independently of grading and tumour stage and may be of additional use for treatment decisions.

## Introduction

Neuroendocrine neoplasms of the pancreas (pNEN) are a heterogeneous group of rare malignancies with increasing incidence^1–4^. The increased usage and quality of imaging techniques, has led to an increase in early stage diagnosis of pNEN at a therefore potentially resectable stage^5,6^. However, prognosis especially in the case of a metastasised situation can be highly variable and is difficult to predict preoperatively.

Surgical resection should always be attempted as a curative treatment option, but the extent of surgical resection is still a matter of debate. Also, in asymptomatic well-differentiated non-functioning (NF)-pNEN smaller than 2 cm, guidelines even recommend surveillance as an alternative^5,6^, although data from big series show that resection of pNEN between 1 and 2 cm does lead to a better survival^7^. However, recent data has shown that the risk of recurrence also in well- (G1) and moderately differentiated (G2) pNEN after surgical resection is variable^8–10^ . Consequently, research has focused on development of risk scores and identification of preoperative risk factors for survival and recurrence in pNEN^11^. Identified preoperative risk factors include functional status, blood glucose, lymph node involvement and distant metastasis^11–18^. Tumour biopsy for grading is also possible, but undergrading and lack of sensitivity are still unsolved^19–23^.

C-reactive protein is an acute-phase protein, which is produced in the liver and is induced in systemic inflammatory responses as a response to stress factors^24^. Preoperatively elevated serum C-reactive protein (CRP) level was identified as a risk factor for poor survival in several tumour entities^25–30^ including surgically resected pancreatic cancer patients^31–34^ and was consequently shown to affect progression in gastroenteropancreatic (GEP-) NEN in general^35^ as well as in pNEN^36^. Only 2 studies have thus far examined the relationship between outcome of surgically resected pNEN and preoperative serum CRP levels^37,38^. However, both studies only analysed the simple presence or absence of elevated serum CRP and did not include grading as one of the most important prognostic parameters in pNEN in the analyses. In both analyses, the observed effect may, therefore, be partially due to the effect of poorly differentiated G3 and advanced tumours. The potential value of CRP as an additional prognostic factor that could guide treatment decisions in pNEN thus remains uncertain.

In this study, we aimed to confirm pre-operative serum CRP as a prognostic factor for outcome and survival after surgical resection of pancreatic NEN independent from grading and tumour advancement. We further aimed to examine whether differences in preoperative serum CRP levels may improve preoperative risk stratification in tumours with given grading. For this purpose, a retrospective analysis on surgically resected pNEN and uni- as well as multivariable analysis for survival with different serum CRP cut-off values was performed.

## Methods

### Patient identification

This study was approved by the ethics committee of the Medical Faculty of the University of Heidelberg (no. S-708/2019) in accordance with the World Medical Association Declaration of Helsinki. All patients undergoing surgical treatment between January 1, 2001 and December 31, 2018 were identified from a prospectively collected neuroendocrine pancreatic database at the Department of General, Visceral and Transplantation Surgery, Heidelberg University Hospital, Germany. Inclusion criteria were pathological diagnosis of a NEN with pancreatic location as well as availability of a pre-operative serum CRP level. The need for informed consent to participate was waived by the ethics committee of the Medical Faculty of the University of Heidelberg due to the retrospective nature of the study.

### Clinical data and surgical approach

Clinical data were additionally extracted from the digital patient information system. The patient cohort was described using the following parameters: age, gender, tumour size, tumour grade based on Ki-67(MIB1) proliferation index in accordance with the WHO classification and the AJCC/UICC classification of malignant tumours^39,40^, TNM stage according to the 8th TNM classification^41^, hormonal status, occurrence of recurrences and pre-operative serum CRP value. CRP levels were measured within 12–36 h prior to surgery.

The following data on the surgical procedure and perioperative period were obtained: intention and type of operation, postoperative complications, extent of lymphadenectomy and 90-day/in hospital mortality. For assessment of post-operative pancreatic fistula (POPF), the international study group classification was used^42^. Postoperative complications were graded according to Dindo et al.^43^.

### Follow-up

Patients were followed until their latest oncologic surveillance examination or until death. Follow-up data were obtained in the outpatient clinic, by external oncologic follow-up, telephone interview of patients, relatives or general practitioners or by information obtained from the residents’ registration offices until January 2020. Follow-up parameters included the current state of disease and in case of death the cause of death.

### CRP assay

CRP was analysed with an immunoassay in the institution’s certified clinical central laboratory with an intra-assay variability of 1.24% and an inter-assay variability of 1.41%.

### Statistical analysis

Statistical analysis was performed using SAS (Release 9.4, SAS Institute, Inc, Cary, NC) and IBM SPSS, version 25 (IBM Corp, Armonk, NY). Quantitative parameters, such as age, length of hospital stay, serum CRP and follow-up, are presented as median and interquartile range. The distribution of continuous data was checked for normality with the Shapiro–Wilk test and comparisons were made with the non-parametric Kruskal–Wallis test. Categorical variables are presented as absolute and relative frequencies and were analysed with the Fisher exact test. Comparisons were performed between three serum CRP groups according to the cut-off values 5 mg/l (upper normal limit of laboratory reference) and 20 mg/l, which has been identified as a cut-off value relevant to survival of pancreatic adenocarcinoma^44^ as well as GEP-NEN^35^.

Overall survival (OS), defined as the time from resection to either death or last follow-up, was analysed using the Kaplan–Meier method and the log rank test in order to compare survival curves in different subgroups. Patients alive at the point of last follow-up were censored. Disease-free survival (DFS), defined as the time from resection to the date of detected recurrence, was also analysed using the Kaplan–Meier method and log rank test. Uni- and multivariable analyses were performed using the proportional hazard regression (Cox model) to assess the relative impact of serum CRP groups on overall survival. The hazard ratios (HR) and their 95% confidence intervals (CI) are presented. Two sided p-values of < 0.05 were considered statistically significant.

## Results

### Patient characteristics and operative data

A total of 559 patients who underwent surgery for a pancreatic NEN at the University Hospital of General, Visceral and Transplantation Surgery, Heidelberg, Germany between 2001 and 2018 were identified. 42 patients, for whom pre-operative serum CRP values were not available, were excluded from the analysis. The remaining 517 patients were included in the analysis (Table 1). Of these 517 patients, 16 (3%) had a segmental pancreatectomy, 86 (17%) underwent enucleation, 236 (46%) patient had a distal pancreatectomy (DP), 19 of which were performed laparoscopically, in 131 (25%) patients partial pancreatoduodenectomy (PD) was performed and 48 (9%) patients underwent total pancreatectomy (TP). In this cohort, 86 of 517 patients (17%) underwent multivisceral operations and 45 of 517 patients (9%) had vascular resections.

**Table 1:** Clinicopathologic characteristics and comparison between CRP groups.

Characteristic			CRP < 5 mg/l** n = 383 (100%)	CRP 5–20 mg/l** n = 97 (100%)	CRP > 20 mg/l** n = 37 (100%)	p value
Age in years, median (IQR)			65 (54–73)	64 (54–73)	69 (60–75)	0.131
Gender	Male		192 (50.1)	52 (53.6)	18 (48.6)	0.802
	Female		191 (49.9)	45 (46.4)	19 (51.4)	
Tumour size in mm, median (IQR)			21 (12–38)	34 (21–50)	36 (21–60)	< 0.001*
Tumour grade^a^ (according to Ki-67 proliferation index; 2 missing values)	G1		227 (59.3)	39 (39.2)	12 (32.4)	< 0.001*
	G2		128 (33.4)	44 (45.4)	12 (32.4)	
	G3		26 (6.8)	14 (14.4)	13 (35.1)	
T status^a^ (8 missing values)	pT1		170 (44.4)	25 (25.8)	9 (24.3)	< 0.001*
	pT2		109 (28.5)	36 (37.1)	8 (21.6)	
	pT3		82 (21.4)	32 (33.0)	20 (54.1)	
	pT4		14 (3.7)	4 (4.1)	0 (-)	
Nodal status^a^ (72 missing values)	pN0		209 (54.6)	46 (47.4)	10 (27)	< 0.001*
	pN1		110 (28.7)	45 (46.4)	25 (67.6)	
Distant metastasis at primary presentation	pM0		336 (87.7)	78 (80.4)	20 (54.1)	< 0.001*
	pM1		47 (12.3)	19 (19.6)	17 (45.9)	
Functioning pNEN	All		62 (16.2)	6 (6.2)	3 (8.1)	0.022*
	Insulinoma		55 (14.4)	4 (4.1)	2 (5.4)	
	Glucagonoma		1 (0.3)	1 (1.0)	0	
	Gastrinoma		4 (1.0)	2 (2.1)	0	
	ACTHoma		1 (0.3)	0	0	
	VIPoma		0	0	0	
	Somatostatinoma		0	0	0	
Recurrence	All		74 (19.3)	28 (28.9)	12 (32.4)	0.037*
	Local recurrence^b^		19 (5.0)	6 (6.2)	3 (8.1)	
	Liver metastasis^b^		46 (12.0)	13 (13.4)	6 (16.2)	
	Distant metastasis other than liver^b^		38 (9.9)	16 (16.5)	8 (21.6)	
	Location of recurrence unknown		6 (1.6)	3 (3.1)	3 (8.1)	

*pNEN* pancreatic neuroendocrine neoplasm, *F-* functioning, *CRP* C-reactive protein.

*Statistically significant.

**Measured 12–36 h prior to surgery.

^a^8th TNM.

^b^Numbers in total, may include patients with both local and distant recurrences.

Major postoperative complications (Clavien-Dindo grade III or higher) were observed in 154 of 517 patients (30%). POPF was observed in 96 of 517 patients (19%), 86 patients with a type B POPF (17%) and 10 patients with a type C POPF (2%). In-hospital mortality was 15 of 517 (3%) patients. 90-day mortality was 18 of 517 (3.5%) patients.

### Clinical differences between serum CRP groups and univariable analysis

The cohort of 517 patients was divided into the three CRP subgroups as explained above with 383 (74%) patients with normal serum CRP values, 97 (19%) patients with serum CRP 5–20 mg/l and 37 (7%) patients with serum CRP values > 20 mg/l. Further analysis was conducted for each subgroup. Clinicopathological characteristics of the study cohort and differences between the three serum CRP groups are shown in Table 1.

For age and gender, there was no statistically significant difference between the three groups. An increase in preoperative serum CRP level was significantly associated with bigger tumours (p < 0.001). Also, higher CRP levels were significantly associated with a higher tumour grade (p < 0.001), higher T status (p < 0.001), positive lymph node status (p < 0.001) and distant metastases (p < 0.001),) reflecting more advanced and aggressive tumours. Interestingly, there was a statistical difference in the amount of functioning pNEN (F-pNEN). Patients with normal serum CRP, had more F-pNEN (62 of 383; 16%) than those with elevated CRP levels with 6 of 97 (6%) serum CRP 5–20 mg/l and 3 of 37 (8%) serum CRP > 20 mg/l (p = 0.022). In F-pNEN, however, the amount of G1 differentiated tumours was significantly higher than the amount of G2 and G3 tumours. The distribution of F- and NF-pNEN was therefore analysed separately for G1, G2 and G3 differentiated tumours and no significant difference was found between the three CRP groups (Table 2). Therefore, F-pNEN were included in all further analyses in order to increase the statistical power.

**Table 2:** Distribution of serum CRP groups according to tumour grade and functioning versus non-functioning pNEN.

Tumour grade (according to Ki-67 proliferation index)	Functioning	CRP < 5 (mg/l) (100%)	CRP 5–20 (mg/l) (100%)	CRP > 20 (mg/l) (100%)	p value
G1	No	183 (80.3)	34 (89.5)	10 (83.3)	0.4374
	Yes	45 (19.7)	4 (10.5)	2 (16.7)	
G2	No	113 (88.3)	42 (95.5)	11 (91.7)	0.3488
	Yes	15 (11.7)	2 (4.5)	1 (8.3)	
G3	No	25 (96.2)	14 (100)	13 (100)	1.0
	Yes	1 (3.8)	0 (0.0)	0 (0.0)	

Importantly, there was a statistically significant increase in observed recurrences the higher the preoperative serum CRP (p = 0.037) both for local recurrences and distant metastases (Table 1).

### Overall and recurrence free survival

Median follow-up time of all 517 patients was 53 months (range: 21–85 months). At last follow-up, 416 (81%) patients were still alive (337 without any evidence of disease), 55 (11%) patients had died due to disease and 37 (7%) patients had died due to other causes, in 5 (1%) patients the cause of death was unknown. 114 (22%) patients had recurrent disease 7 (6%) of 114 patients with local recurrence only, 21 (18%) of 114 local recurrence plus distant metastases, 86 (75%) of 114 with distant metastases without any signs of local recurrence and 19 (4%) patients had progressive disease.

**Figure 1:**
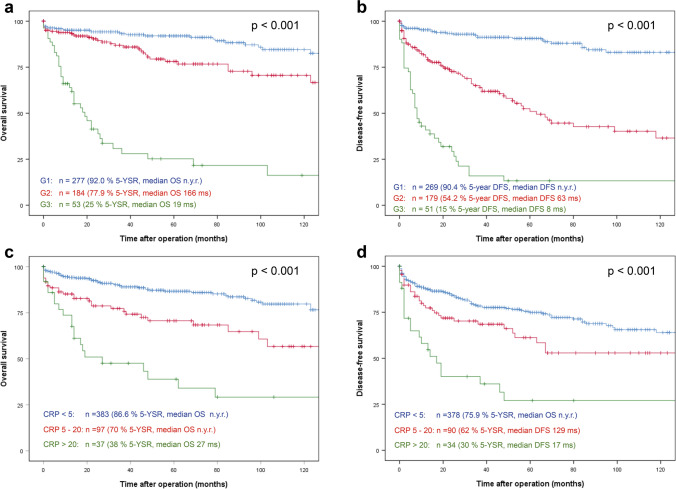
Postresection survival of pNEN stratified by grading and preoperative serum CRP levels. Overall **(a)** and disease-free **(b)** survival of resected pNEN stratified by grading. Overall **(c)** and disease-free **(d)** survival rate of resected pNEN patients stratified by preoperative serum CRP levels: < 5 mg/l, 5–20 mg/l and > 20 mg/l. G1: Ki-67 ≤ 2%; G2: Ki-67 3–20%; G3: Ki-67 > 20%. Patients alive at last follow-up were censored. *5-YSR* 5-year survival rate, *OS* overall survival, *DFS* disease-free survival, *ms* months, *n.y.r.* not yet reached.

For the entire patient population of 517 patients, median overall survival (OS) was not yet reached for G1 differentiated tumours at last follow-up **(**shown in Fig. 1a) and 5-year OS was 92%. In G2 tumours, 5-year OS was 78% (median OS 166 months) and 25% for G3 differentiated tumours (median OS 20 months). 5-year disease-free survival (DFS) was 91% for G1, 50% for G2 and 13% for G3 differentiated tumours (shown in Fig. 1b, p < 0.001). Importantly, there was a highly significant difference in OS of patients stratified by serum CRP. Median OS was not yet reached for patients with normal serum CRP and those with CRP 5–20 mg/l (5-year OS 87% and 70%, respectively), but in patients with serum CRP > 20 mg/l, median OS was 27 months and 5-year OS was 39% (shown in Fig. 1c, p < 0.001). Similarly, DFS was significantly different between the 3 groups with a 5-year DFS of 75% (median DFS not yet reached) and 61% (median DFS 120 months) in patients with normal serum CRP and CRP 5–20 mg/l, respectively and 5-year DFS 30% in patients with CRP > 20 mg/l (median DFS 19 months) (shown in Fig. 1d, p < 0.001).

### Univariable risk factor analysis

As expected, higher age (≥ 70 years), higher T status (T3/T4), positive N status and distant metastases were significantly associated with worse survival in univariable analysis (Table 3). Also, G2 and G3 differentiation of the tumours had a significant impact on survival. Further, a formal resection with PD, DP or TP was significantly associated with survival. Importantly, preoperative serum CRP levels of 5–20 mg/l and CRP levels > 20 mg/l were significantly associated with worse survival (p = 0.0002 and p < 0.0001, respectively).

**Table 3:** Univariable Cox regression analysis of parameters associated with overall survival in n = 517 pancreatic neuroendocrine neoplasms after surgical treatment.

Parameter	Category	N (Deaths)	HR	95% CI	p-value
Gender	Female	255 (33)	1		
	Male	262 (68)	2.12	1.40–3.21	0.0004*
Age at operation	< 70 years	324 (43)	1		
	≥ 70 years	193 (58)	2.07	1.39–3.08	0.0003*
CRP preoperative	< 5 mg/l	383 (52)	1		
	5–20 mg/l	97 (28)	1.20	1.50–3.76	0.0002*
	> 20 mg/l	37 (21)	1.58	3.48–9.64	< 0.0001*
Type of surgery	E/S	102 (2)	1		
	DP	236 (47)	11.14	2.70–45.86	0.0003*
	PD	131 (37)	16.45	3.97–68.28	0.0001*
	TP	48 (15)	17.57	4.02–76.83	0.0001*
Tumor differentiation	G1	277 (28)	1		
	G2	184 (34)	2.15	1.30–3.55	0.0027*
	G3	53 (37)	11.92	7.21–19.70	< 0.0001*
T stage	I/II	357 (39)	1		
	III/IV	152 (59)	3.63	2.42–5.44	< 0.0001*
N stage	LN negative	265 (33)	1		
	LN positive	180 (66)	3.08	2.03–4.69	< 0.0001*
M stage	M0	434 (56)	1		
	M1	83 (45)	5.30	3.57–7.87	< 0.0001*

*HR* hazard ratio, *CI* confidence interval, *CRP* C-reactive protein, *PD* pancreatoduodenectomy, *DP* distal pancreatectomy, *TP* total pancreatectomy, *E/S* enucleation/segmental resection, *LN* lymph node.

*Statistically significant.

### Association of serum CRP with survival in subgroup analysis

**Figure 2:**
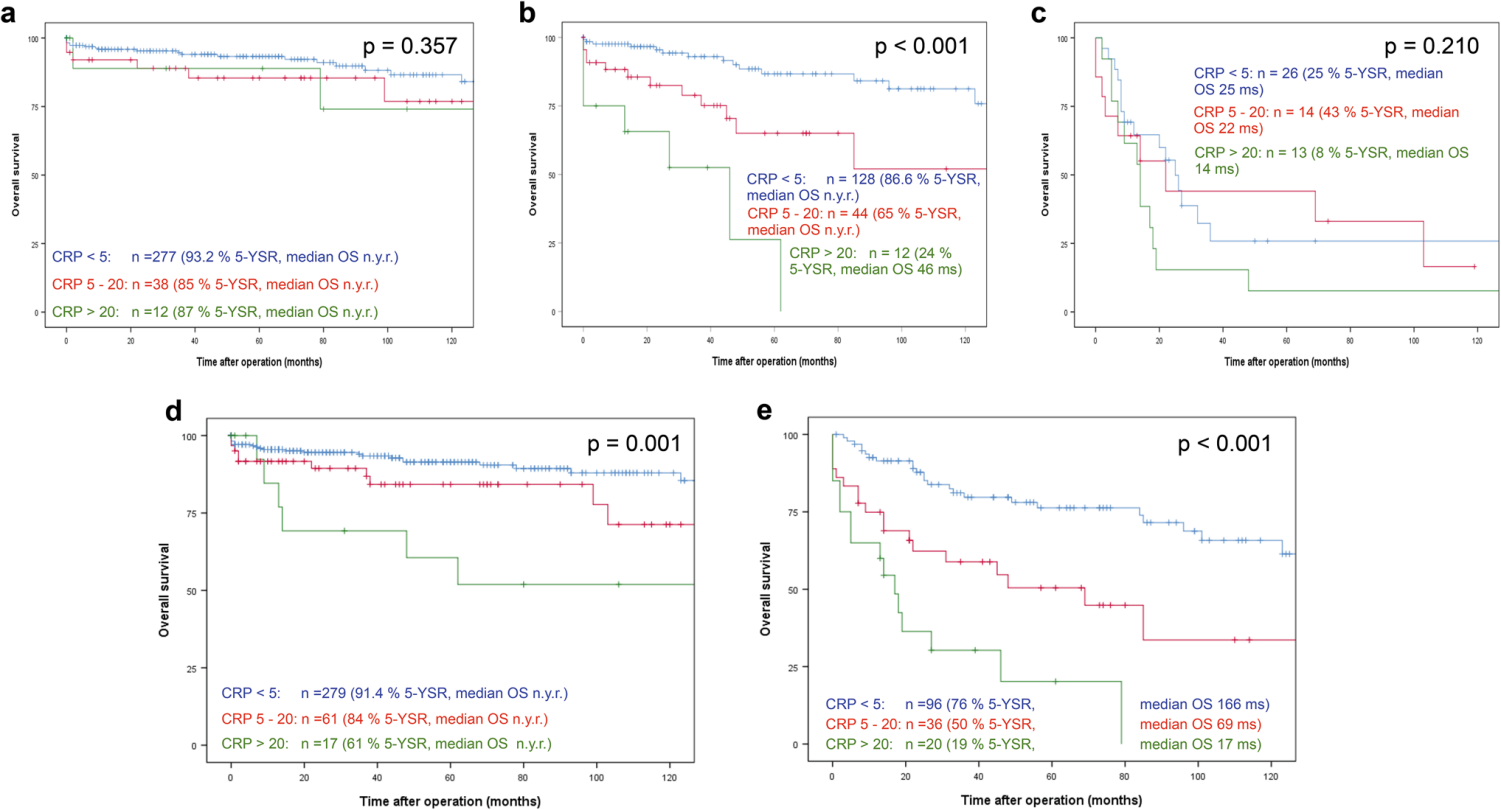
Association of tumour differentiation and stage with survival in serum CRP subgroups. Overall survival rate of resected pNEN patients stratified by serum CRP levels in G1 **(a)**, G2 **(b)** and G3 **(c)** differentiated tumours as well as in pT1 & pT2 **(d)** and pT3 & pT4 tumours **(e).** G1: Ki-67 ≤ 2%; G2: Ki-67 3–20%; G3: Ki-67 > 20%; group 1: CRP < 5 mg/l; group 2: CRP 5-20 mg/l; group 3: CRP > 20 mg/l. Patients alive at last follow-up were censored. *5-YSR* 5-year survival rate, *OS* overall survival, *DFS* disease-free survival, *ms* months, *n.y.r.* not yet reached.

**Figure 3:**
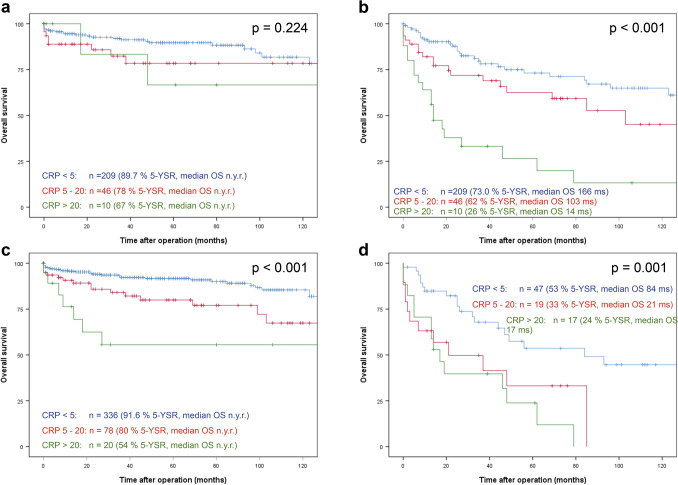
Association of lymph node and distant metastases with survival in serum CRP subgroups. Overall survival rate in resected pNEN patients in node negative **(a)** and node positive patients **(b)** and patients without **(c)** or with distant metastases **(d)** at primary presentation. Group 1: CRP < 5 mg/l; group 2: CRP 5–20 mg/l; group 3: CRP > 20 mg/l. Patients alive at last follow-up were censored. *5-YSR* 5-year survival rate, *OS* overall survival, *DFS* disease-free survival, *ms* months, *n.y.r.* not yet reached.

We further performed subgroup survival analysis within the three grading groups. Importantly, there was a significant difference in OS in G2 differentiated tumours when stratified by CRP (CRP normal: n = 128, 5-year OS 87%—median OS not yet reached, CRP 5–20 mg/l: n = 44, 5-year OS 65%—median OS not yet reached, CRP > 20 mg/l: n = 12, 5-year OS 24%—median OS 46 months; p < 0.001; Fig. 2b). In G1 (CRP normal: n = 277, 5-year OS 93%—median OS not yet reached, CRP 5–20 mg/l: n = 38, 5-year OS 85%—median OS not yet reached, CRP > 20 mg/l: n = 12, 5-year OS 87%—median OS not yet reached; p = 0.357; Fig. 2a ) and G3 (CRP normal: n = 26, 5-year OS 25%—median OS 25 months, CRP 5-20 mg/l: n = 14, 5-year OS 43%—median OS 22 months, CRP > 20 mg/l: n = 13, 5-year OS 8%—median OS 14 months; p = 0.210; Fig. 2c) differentiated tumours, a similar trend could be observed, which was not statistically significant likely due to insufficient statistical power. Subsequent subgroup survival analysis according to T-, N-, and M-status was performed. Pancreatic NEN with a T1 or T2 status (shown in Fig. 2d) and T3 or T4 tumours (shown in Fig. 2e) had a highly significant difference in survival stratified by serum CRP (p = 0.001 and p < 0.001, respectively). Median OS was not yet reached for T1 and T2 tumours. In T3 and T4 tumours median OS was 166 months, 69 months and 17 months for normal serum CRP values, CRP 5-20 mg/l and CRP > 20 mg/l, respectively. With regards to nodal status (pN), overall survival stratified by CRP in node positive (pN1) patients (shown in Fig. 3b) was significantly different with a 5-year OS of 73% (median OS 166 months), 63% (median OS 103 months) and 26% (median OS 14 months) in patients with normal CRP, CRP 5-20 mg/l and CRP > 20 mg/l, respectively (p < 0.001). In node negative patients (pN0) (shown in Fig. 3a), a similar but not significant trend could be observed. Further, survival stratified by preoperative serum CRP also showed highly significant differences both in patients with (c/pM1) and without (pM0) metastasis at primary presentation. Median OS was not yet reached in patients with pM0 status:: 5-year OS was 92%, 80% and 56% in patients with normal CRP, CRP 5–20 mg/l and CRP > 20 mg/l, respectively (p < 0.001, shown in Fig. 3c). In pM1 patients 5-year OS was 53% (median OS 81 months), 33% (median OS 21 months) and 24% (median OS 17 months) in CRP normal, 5–20 mg/l and > 20 mg/l, respectively (p = 0.002, shown in Fig. 3d). These results indicate that the observed clinically highly significant differences in survival when stratified by CRP do not result from inhomogeneous occurrences of other known prognostic factors. In order to confirm CRP as an independent prognostic factor, multivariable analysis was performed.

### Multivariable risk factor analysis

In multivariable analysis, the preoperative risk factors independently predicting postresection survival of pNEN were age and serum CRP. The latter proved a significant impact on survival with an HR of 1.65 and 2.41 for normal serum CRP versus CRP 5–20 mg/l and versus CRP > 20md/l, respectively (Table 4). Known postresection factors that are independently associated with postresection survival, such as poor differentiation (G3), T stage and metastatic status were also confirmed, further validating the performed analysis. The type of surgery, N stage and gender were not included in the multivariable analysis.

**Table 4:** Multivariable Cox regression analysis of parameters associated with overall survival in n = 437 pancreatic neuroendocrine neoplasms after surgical treatment (n = 80 patients with missing values were excluded).

Parameter	Category	HR	95% CI	p-value
(Likelihood ratio: Chi^2^ 139.31, 12 DF, p < 0.0001)				
Tumour differentiation	G2 vs. G1	1.46	0.85–2.49	0.1697
	G3 vs. G1	5.97	3.38–10.55	< 0.0001*
M stage	M1 vs. M0	2.75	1.72–4.42	< 0.0001*
Age at operation	≥ 70 years vs. < 70 years	2.67	1.74–4.08	< 0.0001*
CRP preoperative	5–20 mg/l vs. < 5 mg/l	1.65	1.01–2.72	0.0477*
	> 20 mg/l vs. < 5 mg/l	2.41	1.38–4.19	0.0020*
T stage	III/IV vs. I/II	1.64	1.02–2.64	0.0433*
**Not included**				
Type of surgery	TP vs. PD vs. DP vs. E/S			0.6242
N stage	LN positive vs. LN negative			0.2323
Gender	Male vs. female			0.0741

*HR* hazard ratio, *CI* confidence interval, *CRP* C-reactive protein, *PD* pancreatoduodenectomy, *DP* distal pancreatectomy, *TP* total pancreatectomy, *LN* lymph node, *E/S* enucleation/segmental resection.

*Statistically significant.

The multivariable analysis was repeated for the 395 NF-pNEN patients only with similar results and therefore confirmed CRP as an independent risk factor for survival (Table 5).

**Table 5:** Multivariable Cox regression analysis of parameters associated with overall survival in n = 395 non-functioning pancreatic neuroendocrine neoplasms after surgical treatment (n = 51 patients with missing values were excluded).

Parameter	Category	HR	95% CI	p-value
(Likelihood ratio: Chi^2^ 120.10, 7 DF, p < 0.0001)				
Tumour differentiation	G2 vs. G1	1.27	0.74–2.20	0.3902
	G3 vs. G1	5.51	3.09–9.82	< 0.0001
M stage	M1 vs. M0	2.48	1.52–4.04	0.0003
Age at operation	≥ 70 years vs. < 70 years	2.54	1.64–3.94	< 0.0001
CRP preoperative	5- < 20 mg/l vs. < 5 mg/l	1.61	0.96–2.70	0.0715
	≥ 20 mg/l vs. < 5 mg/l	2.59	1.47–4.55	0.0010
T stage	III/IV vs. I/II	1.80	1.10–2.95	0.0204
**Not included**				
Type of surgery	TP vs. PD vs. DP vs. E/S			0.8203
N stage	LN positive vs. LN negative			0.1891
Gender	Male vs. female			0.1075

*HR* hazard ratio, *CI* confidence interval, *CRP* C-reactive protein, *PD* pancreatoduodenectomy, *DP* distal pancreatectomy, *TP* total pancreatectomy, *E* enucleation, *S* segmental resection, *LN* lymph node.

### Receiver operating curve

**Figure 4:**
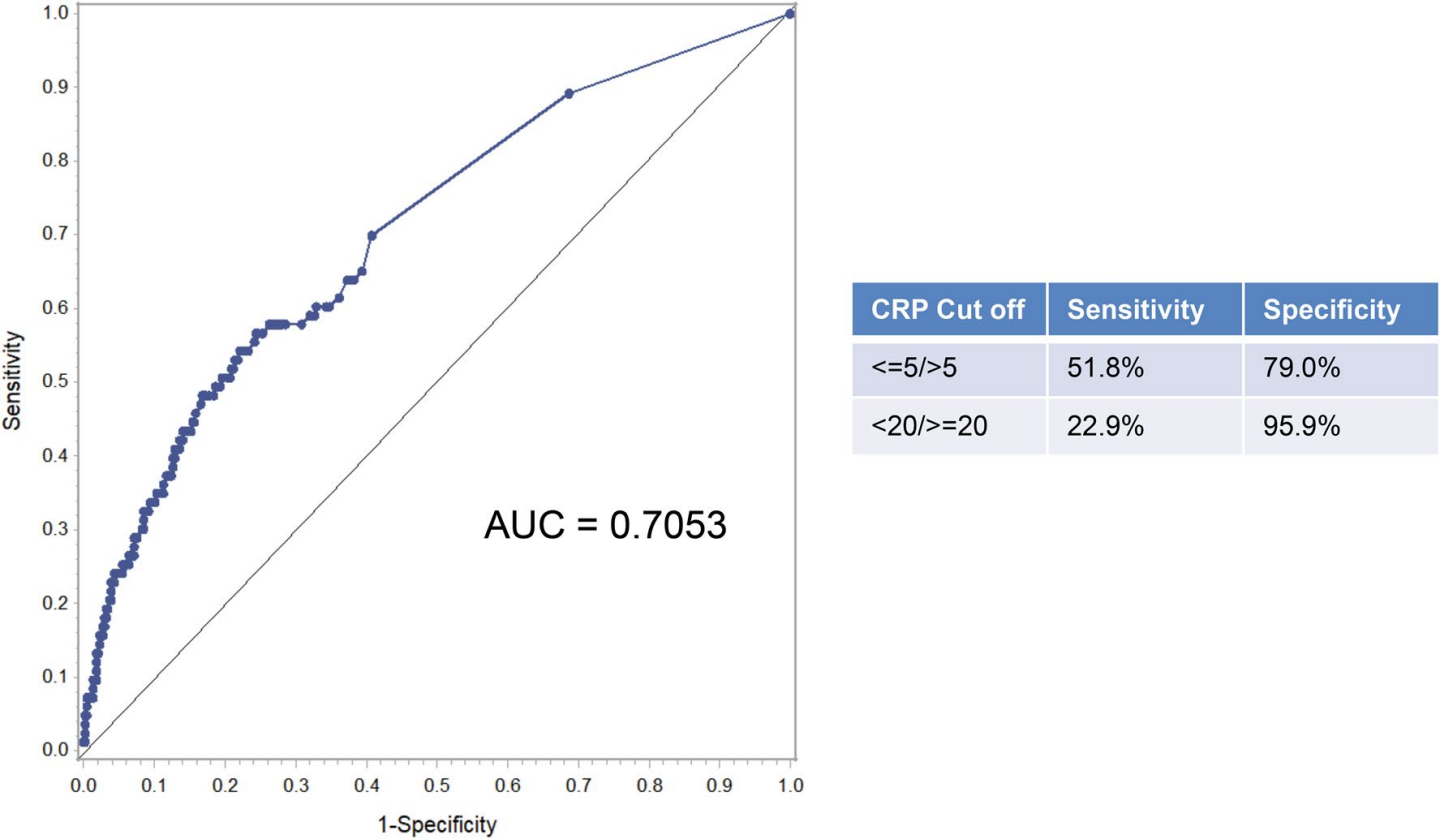
ROC curve analysis of preoperative serum CRP at 5 year follow-up. *CRP* C-reactive protein (mg/l), *ROC* receiver operating characteristic, *AUC* area under curve.

Receiver operating curve (ROC) analysis (Fig. 4) was performed. For CRP 5 mg/l sensitivity (SE) was 52% and specificity (SP) 79%, for 20 mg/l SE was 23% and SP 97%.

## Discussion

Serum CRP has been identified as a major player in prediction of survival in several tumour entities^25,29,30,45^. Elevated CRP levels as part of tumour-associated inflammation is a well-known phenomenon. As such, inflammation scores including CRP, such as the Glasgow inflammation score (GPS)^46^, have been shown to correlate significantly with survival in different tumour entities^44,47,48^. In 2016, Wiese et al., for the first time identified serum CRP also as an independent prognostic factor for survival in pNEN patients^37^. In their study of 149 surgically resected pNEN, pre-operatively elevated serum CRP (> 5 mg/l) was significantly associated with poorer outcome in comparison to those with normal serum CRP values and was confirmed as an independent risk factor for overall survival in multivariable analysis. In their study, however, grading as a well-established postresection prognosis factor for survival^49^ was not taken into consideration. The authors did examine the relationship between Ki-67 staining < 5% and ≥ 5% and CRP, but did not find a correlation. Also, Ki-67 < 5% and ≥ 5% was not confirmed as an independent risk factor for overall survival in multivariable analysis. In contrast, the current analysis not only showed that stratification of postresection survival of pNEN is similar for grading as a postresection risk factor and serum CRP as a preoperative risk factor (shown in Fig. 1), but also confirms preoperative serum CRP as an independent preoperative risk factor associated with overall survival in multivariable analysis independent from grading and advanced tumour stages (Tables 3 and 4).

Similarly, Primavesi et al.^38^ analysed 364 surgically resected patients in a retrospective multicentre study. The authors report significantly higher serum CRP levels in older patients (≥ 60 years), tumours ≥ 3 cm and G3 differentiated patients. They further show a significant difference in OS and DFS in serum CRP low (< 0.2 mg/dl) versus high (≥ 0.2 mg/dl) and also confirm CRP as an independent risk factor for overall survival in multivariable analysis. The serum CRP value, however, is as stated by the authors well below the lower reference limit of most laboratories. The reference limits, which are likely to differ between the different centres’ laboratories, are not stated. The clinical usage of these cut-off values is thus limited. The authors also suggest that high serum CRP levels are associated with more aggressive tumour characteristics such as larger tumour size and less differentiated tumours (G3). In their analysis, tumour differentiation is not included in the multivariable analysis at all. The observed effect, may therefore be biased by advanced tumour stages and poor differentiation of the tumour.

In this study, we further demonstrated that different preoperative serum CRP levels may be used for preoperative risk stratification of pNEN patients. The survival curves for patients with different serum CRP levels (shown in Fig. 1c) show similar patterns as those observed in patients with different tumour grading (shown in Fig. 1a)^50,51^, which is the main variable for the WHO-classification of neuroendocrine disease^39,40^ and implemented in the European Neuroendocrine Tumour Society (ENETS) guidelines^5^. From the data by Primavesi et al.^38^, one might argue that the same biological effect is measured. Importantly, however, serum CRP is shown to be a risk factor independent of grading in this analysis, thereby disproving this hypothesis. Furthermore, the data by Schimmack et al. provide arguments that serum CRP is not measuring the same phenomenon as Ki-67 by showing the (autocrine) impact of CRP on tumour cells^36^.

Our analysis shows that preoperative serum CRP levels correlate with overall survival independent from grading. However, in G3 differentiated pNEN for example, 5-year overall survival was only 25% for patients with normal CRP (Fig. 3c). Thus, preoperative serum CRP levels alone do not serve as a factor that allows for stratification of a surveillance group. It may, however, serve as a prognostic factor for postresection management of these patients.

Within the cohort of 517 surgically resected pNEN patients, age was not significantly different between the three CRP groups (Table 1, p = 0.131). As the univariable (Table 3) and multivariable analysis (Table 4) are performed with regards to the overall postresection survival of pNEN patients, naturally older patients have a worse overall survival due to increased comorbidities and a statistically increased likelihood of passing. Importantly, however, the multivariable analysis confirms that higher preoperative serum CRP levels pose a risk factor for worse overall survival after surgical resection of a pNEN independent from the age of a patient (Table 4).

In terms of ROC analysis (Fig. 4), it is important to note that in the current analysis, the preoperative serum CRP level was evaluated as a prognostic factor and confirmed as an independent prognostic factor for overall survival in resected pNEN. Thus, this is not to be equalised to a preoperative diagnostic tool that naturally demands for the highest SE and SP possible.

This study, for the first time, demonstrates that preoperative serum CRP is an independent risk factor for postresection survival regardless of tumour differentiation and tumour advancement. Also, the level of preoperative serum CRP levels may serve to facilitate preoperative risk assessment and help to improve informed treatment decisions, especially in borderline decisions for well- and intermediately differentiated tumours.

The main limitation of this study is its retrospective nature. The data may also be subject to some bias based on referral and selection of patients for surgery. Exclusion of infection as a contributing factor to the elevated serum CRP level cannot be provided for every patient. However, all patients were operated on in an elective setting. At our clinic, any patient scheduled for an elective operation presenting with a clinically relevant increase in preoperative CRP level (> 20 mg/l) is checked for general infections (fever, shivering, sore throat, poor general health) as well as for urinary and pulmonary tract infections through urine analysis and chest x-ray. In case of infection, those patients are not admitted to elective surgery.

Considering the available evidence, we advocate serum CRP being an obligate factor for risk stratification in pNEN.

## Conclusion

In spite of the limitations, this is the largest single-centre study reporting on the value of pre-operative serum CRP levels. This study demonstrates that a pre-operatively elevated serum CRP level in surgically resected patients is an important prognostic factor for survival independent from tumour differentiation and tumour stage. Different serum CRP levels may serve as a basis for prognostic stratification, especially in moderately differentiated (G2) tumours. Importantly, in contrast to grading, serum CRP is a reliable and easily available laboratory marker that can be determined in almost any pNEN patient prior to surgery at low cost. It is the first study showing that not only elevation, but different serum CRP cutoff levels are highly prognostic in surgically resected pNEN patients. As serum CRP levels 5–20 mg/l and > 20 mg/l were confirmed as independent risk factors for worse outcome in multivariable analysis, the two cut-off values (< 5 mg/l and > 20 mg/l) allow for prognostic stratification of pNEN patients depending on their preoperative serum CRP level. We propose that serum CRP might be a factor worth evaluating for additional prognostic information of the ENETS or WHO guidelines and staging systems for pancreatic NEN.
